# *In vitro* anti-proliferative effect of capecitabine (Xeloda) combined with mocetinostat (MGCD0103) in 4T1 breast cancer cell line by immunoblotting

**DOI:** 10.22038/IJBMS.2021.58393.12971

**Published:** 2021-11

**Authors:** Hacer Kaya Çakir, Onur Eroglu

**Affiliations:** 1 Department of Molecular Biology and Genetics, Faculty of Science and Letters, Bilecik Seyh Edebali University, Bilecik, Turkey; 2 Biotechnology Research and Application Center, Bilecik Seyh Edebali University, Bilecik, Turkey

**Keywords:** Apoptosis, Breast neoplasms, Drug synergism, Capecitabine, Histone deacetylase - inhibitors

## Abstract

**Objective(s):**

Mouse breast cancer cell line 4T1 can accurately mimic the response to immune receptors and targeting therapeutic agents. Combined therapy has emerged as an important strategy with reduced side effects and maximum therapeutic effect. Mocetinostat (MGCD0103) is one of the members of Class I Histone Deacetylase Inhibitors (HDACi) and its mechanism of action has not been defined, yet. Capecitabine (Xeloda) is an antimetabolite and currently is widely utilized to treat a wide range of solid tumors. The aim of this study was to investigate the effects of the capecitabine, mocetinostat and their combined application on the 4T1 cell line.

**Materials and Methods:**

The effects of combined administration of mocetinostat and capecitabine on 4T1 cells were investigated by cell viability and migration assays, apoptosis analysis, and Western blotting technique.

**Results:**

The concentrations of drugs that give a half-maximal response (IC_50_) were detected for capecitabine (1700 µM), mocetinostat (3,125 µM), and 50 µM Capecitabine+1,5 µM Mocetinostat for 48 hr. In capecitabine+mocetinostat combine group, we observed that cell migration decreased, DNA fragmentation increased compared to the control group. capecitabine + mocetinostat group induced apoptosis by decreasing Bcl-2, PI3K, Akt, c-myc protein levels, while increasing Bax, Caspase-3, PTEN, cleaved-PARP, Caspase-7, Caspase-9, p53, cleaved-Cas-9 protein levels in 4T1 cells.

**Conclusion:**

Capecitabine and mocetinostat played a toxic role through inducing apoptosis on 4T1 cancer cells in a time- and concentration-dependent manner. These results showed that combined therapy with low concentrations were detected to be more effective than that with high-concentration alone drug treatment.

## Introduction

TNBC (triple-negative breast cancer) is defined by the absence of estrogen, progesterone, and human epidermal growth factor 2 receptors which account for 15% to 20% of all breast cancer cases (1). TNBC is a heterogeneous subtype of breast cancer whose molecular characteristics and clinical response to a targeted therapeutic approach are beginning to refine it (2). The disease also has a more severe profile than hormone receptor-positive tumors, with higher relapse rates and short life expectancy. 

One of the mechanisms preventing cancer formation and development is apoptosis. The formation of apoptosis is inhibited as a result of tumor cells, disruption of the balance of pro-apoptotic and anti-apoptotic proteins, decrease in caspase activity, and disruption of death receptor signals (3). The Apoptosis mechanism is still dominant in breast cancer today (4). In addition, understanding the apoptotic mechanisms of drugs used in cancer treatment provides very important information on how to treat the disease.

Chemotherapy is a commonly used treatment for breast cancer (5). When cancer drugs are given together, they are more effective in therapy. Combination therapy aims to use drugs that function through various pathways, reducing the chances of cancer cells developing resistance. When drugs with different effects are combined, each of them can be used at its maximum effectiveness without causing intolerable side effects (6-7). Two or more therapeutic proposals specifically target the cancer-causing cell or signaling pathways, gaining the advantage of reaching multiple targets in determining the different mechanisms of drugs. Combination therapy is gaining traction as a viable technique for achieving a better long-term prognosis with fewer side effects and maximum therapeutic efficacy (8). This combination therapy is currently being tested in clinical trials as a powerful new cancer treatment technique (9).

The epigenetic mechanism is known to be involved in the initiation and progression of TNBC. As a result, the mechanisms, molecules, and signaling pathways of genes that act in and express epigenetic regulation in carcinogenesis are gaining attention (10). HDACi have been shown to inhibit tumor development, induce apoptosis, and control cellular functions ranging from metastasis to angiogenesis in cancer cells. Furthermore, HDACi have been shown to cause significantly less cytotoxicity in normal cells (11). Mocetinostat is a class I and IV selective HDACi that has been shown in preclinical studies to have potent and selective antiproliferative effects in a variety of human cancer cells (12). Mocetinostat is well-tolerated in clinical trials, with favorable pharmacokinetics and pharmacodynamics and promising antitumor activity in many hematological diseases (13). Another effective strategy for the treatment of solid tumors is to combine mocetinostat with other antitumor agents (14).

Capecitabine is an anti-cancer chemotherapeutic. It is classified as an antimetabolite. Capecitabine was formed based on observation of a high concentration of thymidine phosphorylase enzyme in many human tumors, and it has low toxicity and is easy to administer. It acts throughout the S phase of the cell cycle by inhibiting DNA synthesis through restricting the availability of thymidylate and inducing apoptosis in cells (15).

In this study, mocetinostat and capecitabine were applied in combination to 4T1 breast cancer cells for the first time and combined use of capecitabine and mocetinostat were evaluated comparatively. The viability and apoptosis of cancer cells were identified at the cellular and molecular levels in order to obtain a more detailed and mechanistic understanding of the toxic effects of combined treatment with mocetinostat and capecitabine on 4T1 cancer cells. 

## Materials and Methods

***Preparation of mocetinostat and capecitabine***

Mocetinostat (MedChem, USA) and Capecitabine (MedChem, USA) were dissolved in dimethyl sulfoxide (DMSO) at 100 mM and stored at −20 °C. The concentrations used in this study were: 1,625, 3,125, 6,25, 12,5, and 25 µM for mocetinostat (12) and 800, 900, 1000, 1200, 1400,1600, 1700, 1800, and 2000 µM for capecitabine (29) freshly diluted in DMEM F12 medium before use. Untreated cells were used as a control group. Also, DMSO was used as a control group medium prepared with 1% DMSO.

***Cell line and reagents***

4T1 breast cancer cell line provided by Assoc. Dr. Ayşe NALBANTSOY and was cultured in Dulbecco’s modified Eagle’s medium F12 (DMEM F-12); Gibco, New Zeland) with 10% fetal bovine serum (FBS; Gibco, New Zeland), 100 U/ml penicillin and 100 μg/ml streptomycin (Invitrogen, USA) in a humidified atmosphere of 5% CO2 at 37 °C. Antibodies used for Western blot analysis: Caspase 3 (Cell Signaling Technology-9662, Rabbit, Monoclonal), Caspase 7 (Cell Signaling Technology- 12827, Rabbit, Monoclonal), Caspase 9 (Cell Signaling Technology- 9508, Rabbit, Monoclonal), Bcl-2 (Santa Cruz -7382, Mouse, Monoclonal), Bax (Santa Cruz -20067, Mouse, Monoclonal) C-PARP, c-myc (Santa Cruz - 40, Mouse, Monoclonal), pten (Santa Cruz -7974, Mouse, Monoclonal), p53 (Santa Cruz -126, Mouse, Monoclonal), AKT (Santa Cruz -5298, Mouse, Monoclonal), PI3K (Santa Cruz -1637, Mouse, Monoclonal), Hdac1 (Santa Cruz -81598, Mouse, Monoclonal), HdacIII (Santa Cruz -376957, Mouse, Monoclonal) β-actin (47778 were purchased from Santa Cruse Biotecnology. The secondary antibody (Bioassay Tecnology-AP05855, Rabbit, Polyclonal/Santa Cruz -7076, Mouse, Polyclonal) and RIPA lysis buffer were bought from Cell Signaling Technology-9806, Inc (Boston, USA). 

***Cell viability assays***

The effects of mocetinostat, capecitabine, and capecitabine+mocetinostat on the viability of 4T1 cells were analyzed using the MTT assay. 4T1 cells were seeded at a density of 6000 cells per well in a 96-well plate and incubated for 24 hr before being treated with vehicle (DMSO at a final concentration of 0.5%), mocetinostat, capecitabine, and capecitabine+mocetinostat. Following a 48-hour incubation period, the MTT test was carried out according to the manufacturer’s instructions (Acros Organics, China). The absorbances were read at 570 nm with a microplate reader (ThermoFisher Scientific) and the mean values were calculated based on the data of three independent replicates. The concentration and % cell viability curve applied with the help of the Microsoft Excel program were calculated using the formula

Y = mx + C (16)

(Y = inhibition, x = concentration, C = constant, m = coefficient).

***Cell morphology analysis***

4T1 cells were seeded into 6-well plates at a density of 1 × 10^3^ cells in each well and incubated for 24 hr in fresh media. The culture medium was then replaced with a freshly prepared culture medium, and all of the drugs were given at the concentration of their IC_50_ value, after which they were incubated for 0, 24, 48, and 72 hr in the same setting. The culture media was collected after incubation, and the cells were rinsed in PBS (pH 7.4) and examined under an inverted microscope (Nikon Eclipse TS100).

***Trypan blue dye exclusion assay***

Trypan Blue technique is one of the measures of cells’ metabolic status markers or their ability to perform complex metabolic tasks. 4T1 cancer cells were seeded 1x10^5 ^cell/ml in 6-well plates and the cells were treated with drugs at 24, 48, 72, and 96 hr according to the IC_50_ values obtained for the drugs. After trypsinization, 0.5% trypan blue solvent was added and cells were counted with a hemocytometer. 

***DNA fragmentation analysis***

DNA fragmentation assay was performed using the agarose gel electrophoresis method previously mentioned to confirm cell death via apoptosis (17). In brief, 4T1 cells (1×10^6^) were seeded in T75 flasks and incubated for 24 hr. The cells were then treated with different concentrations of mocetinostat (3,125 µM), capecitabine (1700 µM), capecitabine+mocetinostat (1,5 µM+50 µM) and incubated again for 48 hr. Then, the cells were washed in PBS. Total DNA was isolated and analyzed by electrophoresis on 2% gel containing 0.1 μg/ml of ethidium bromide and visualized under a UV illuminator.

***Cell wound healing assay***

The ability of live cells to migrate is essential for normal growth, immune response, and disease processes including cancer metastasis and inflammation. 4T1 cells (1 × 10^5^) were seeded into a 6-well plate and cultured in a complete medium. When the cells had reached 75% confluence, the cell layers were damaged using a sterile pipette tip and incubation continued with mocetinostat, capecitabine, capecitabine+mocetinostat for 72 hr. The *in vitro* healing mechanism refers to the movement of cells through the wound surface. An inverted microscope was used to photograph the wound healing ın vitro and measure the rate of closure.

The rate of wound healing = [(the wound width of 0 hr – 48 hr)/ 0 hr wound width] × 100% (18).

***SDS-PAGE and Western blot analysis***

4T1 cells were collected by 1XPBS and centrifuged for 2 min at 13,200 rpm. After harvesting, cells were extracted with radioimmunoprecipitation assay (RIPA) lysis buffer added Phenyl-methyl-sulfonyl-fluoride (PMSF) to protect proteins from degradation. The samples were incubated for 20 min at room temperature before being centrifuged at +4 °C for 20 min at 13,200 rpm. The protein concentration was determined using the Bradford assay and protein samples were separated by SDS-PAGE with equal amounts of total protein (50 μg) gel electrophoresis using 12% polyacrylamide gels. SDS-PAGE was used to separate the lysates, which were then transferred to polyvinylidene difluoride (PVDF) membranes. Ponceau Red staining was used to track protein transfer. 5% skim milk in Tris-buffered saline (TBS) containing 0.1% Tween -20 (TBST) solution was used to block membranes for 1 hr. Primary antibodies: Bcl -2, Bax, c- myc, caspase 7, caspase 3, caspase 9, Hdac I, Hdac III, PTEN, Cleaved PARP, p53, Akt(1:1000), PI3K, HdacI, and HdacIII were added and incubated at a temperature of 4 °C, overnight. Each membrane was washed five times for 5 min using TBST and was then incubated with appropriate secondary antibodies for 2 hr on a shaker at room temperature. Protein signals were detected using enhanced chemiluminescence (ECL). The densitometry of immunoblots was quantified with Image J software.

***Statistical analysis***

All data were expressed as mean ± standard deviation (SD). Multiple group comparisons were performed by one-way analysis of variance (ANOVA) prepared in GraphPad Prism 9.1.1. “ImageJ application” program is used for the density measurement of the monitored bands. Each protein band had been measured three times and mean value of three measurement had been used. For the calculation of relative expression levels; each value had been divided to values of β-actin. 

## Results

***Cell viability assays***

**Figure 1:**
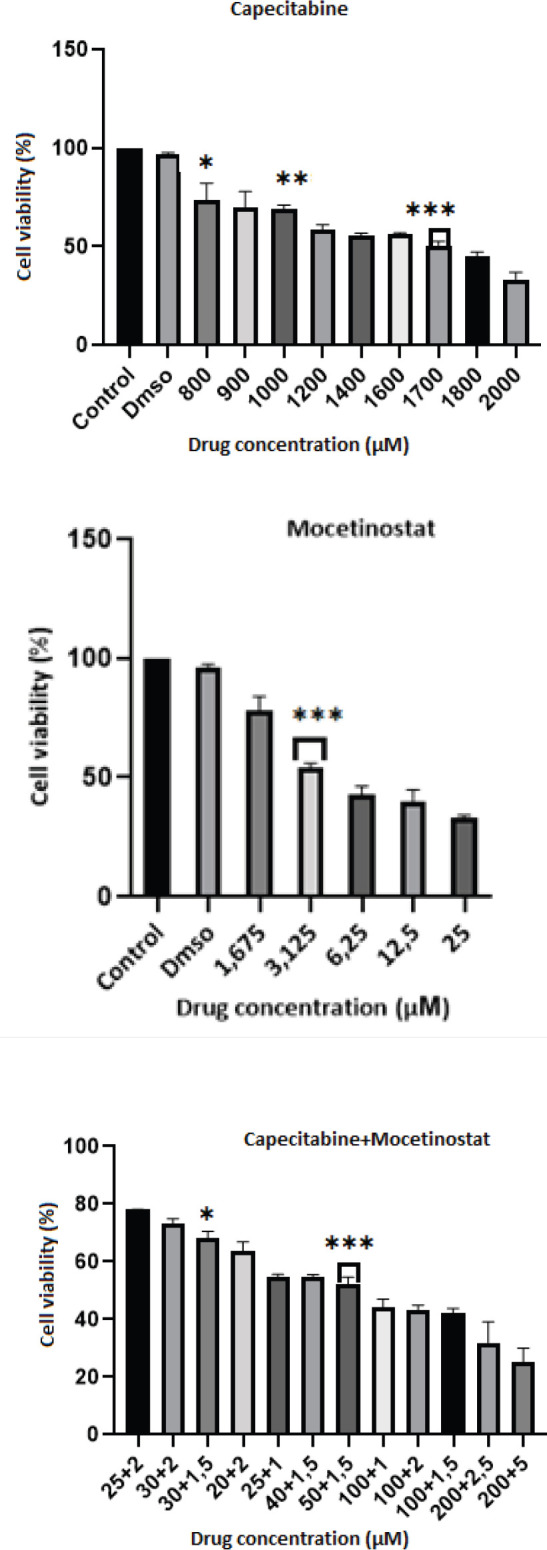
Effect of capecitabine, mocetinostat, and capecitabine+ mocetinostat on 4T1 breast cancer cell viability for 48 hr. The effect of drugs on the survival rate of 4T1 cells was quantified by the MTT method. The data are shown as the mean SD of three separate experiments, with error bars denoting SD. *P< 0.05, **P< 0.01, ***P< 0.001 when compared with untreated cells

The potent inhibitory effects of capecitabine, mocetinostat, capecitabine+ mocetinostat time-dependency on 4T1 cell viability were showed in Figure 1. IC_50_ values obtained from MTT results were determined as 1700 µM for capecitabine, 3,125 µM for mocetinostat, 50 µM+1,5 µM for capecitabine+ mocetinostat combine treatment (1,5 µM+50 µM). 

***Cell morphology analysis***

**Figure 2:**
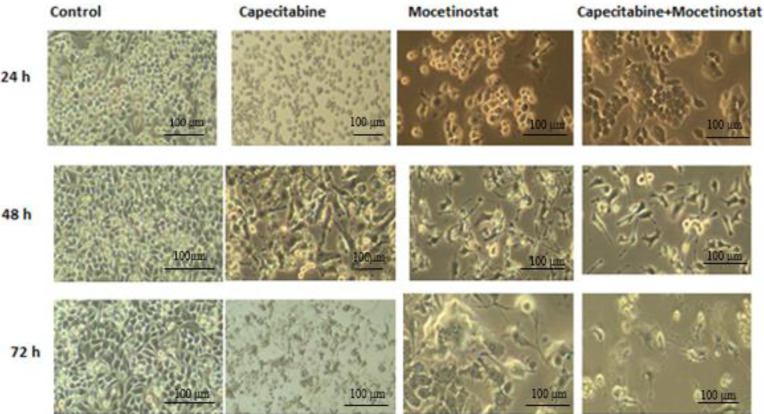
Time-dependent morphological image of 4T1 cells treated with mocetinostat capecitabine and combination of two drugs (Nikon Eclipse TS100)

4T1 breast cancer cells are shown with inverted microscope images in Figure 2. Treatment of capecitabine, mocetinostat, capecitabine+mocetinostat cells appears to cause abnormal changes, such as condensation in the cell nucleus, reduced cell density and number, reduction in cell size and losing cell extensions, and turning into a round shape. These changes in 4T1 cells showed the effects of drugs at different times and different concentrations.

***Trypan blue dye exclusion assay***

**Figure 3:**
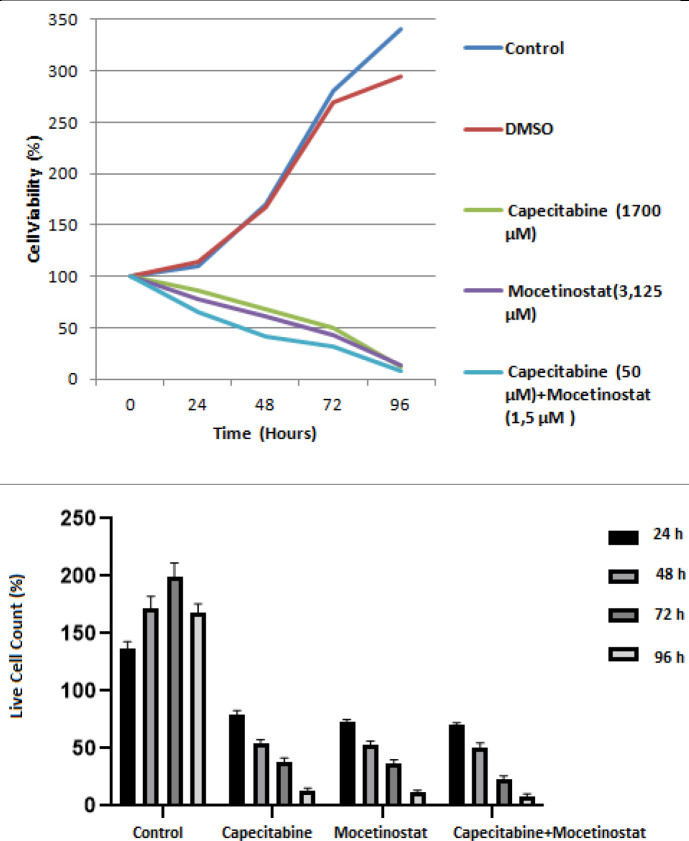
Time dependency of the viability of 4T1 breast cancer cells after capecitabine, mocetinostat, and capecitabine+mocetinostat treatment

The drug-treated and control group’s morphological characteristics at 24, 48, 72, and 96 hr after the 4T1 cell line was cultured are seen in Figure 3. Depending on the time, drug-treated cell groups have a substantial reduction in cell proliferation. The rates of dead and living cells were calculated as a result of the counts, and it was shown that capecitabine caused increased lethal effect at 24%, 45.2%, 69.1%, and 86.6%; mocetinostat caused 28%, 42%, 61.5%, and 77.8% increased lethal effect; capecitabine+mocetinostat caused 16%, 34.3%, 51.5%, and 88.2 % increased lethal effect, respectively for 24, 48, 72, and 96 hr compared with the control group.

***DNA fragmentation analysis***

**Figure 4:**
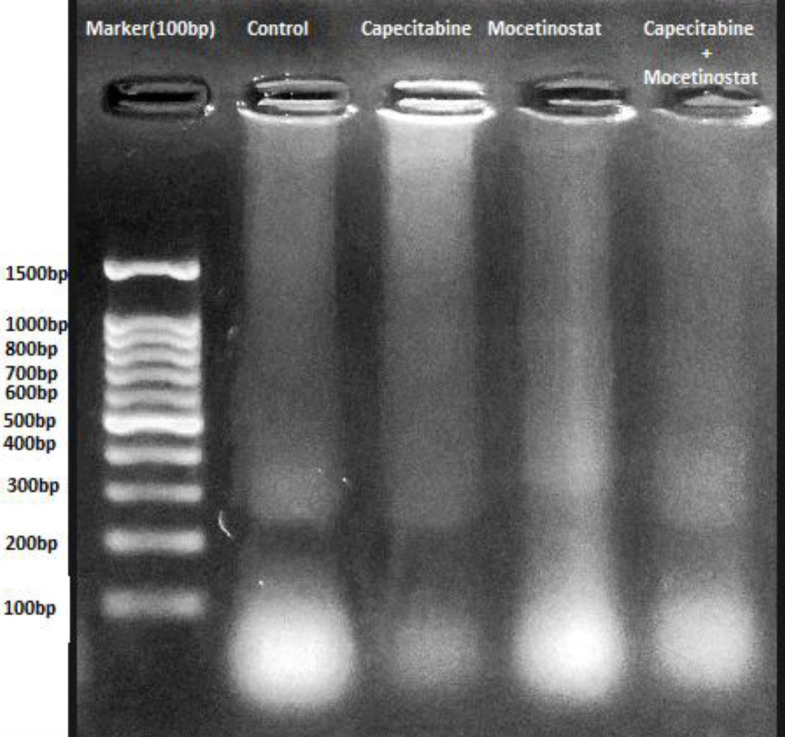
DNA laddering was performed in the control and treatment groups in an agarose gel to support the induction of apoptosis by capecitabine, mocetinostat, and capecitabine+mocetinostat for 48 hr

Apoptosis is characterized by the fragmentation of DNA. 4T1 cells (Figure 4).

Treated with 1700 µM capecitabine, 3,125 µM mocetinostat, and 50 µM capecitabine+1,5 µM mocetinostat. DNA fragmentation was seen in a time-dependent manner with both high molecular weight DNA. The intact DNA bands were seen in the control group, which was treated with 0.1 % DMSO. DNA laddering was also observed in cells treated with drugs as a supportive influence. The treated cells had a DNA laddering pattern. 

***Cell wound healing assay***

In the wound healing experiment, 4T1 breast cancer cell migrations following 24 hr, 48 hr, and 72 hr after capecitabine, mocetinostat, capecitabine +mocetinostat treatment the closure of the width of the wound was examined at 24-hr intervals and the measurement results were recorded in triple repetition. The migration rate of drug-treated cells was decreased compared with the control groups. The control group’s wound width was recorded as an average of 851,7 μm at the 0th hour and was completely closed at the end of the 72nd hour. Control was 456,05 μm, capecitabine 866,50 μm, mocetinostat 825,17 μm, and capecitabine+mocetinostat 856,14 μm at 48 hr. These results were evaluated as decreased cell proliferation and increased wound width by drug treatment in a time-dependent manner in 4T1 cells.

***Western blot analysis***

Effects of capecitabine, mocetinostat, and capecitabine+mocetinostat on the apoptotic signaling pathway in 4T1 cells were verified by Western blotting analysis, subsequent to the cells being treated with capecitabine 1700 µM, mocetinostatat 3,125 µM and capecitabine 50 µM+mocetinostat 1,5 µM for 48 hr. Following treatment, Western blot analysis showed that expression of Bcl2, Hdac I, Akt, PI3K, c-myc, and Hdac III proteins were significantly decreased, and Bax, Cas-3, Pten, C-Parp, Cas-7, Cas-9, p53, and C-cas9 protein expression was significantly increased by capecitabine and mocetinostat treatment (significant differences: * *P*<0.05, ** *P*<0.01, ***P<0.001, Figure 6). 

**Figure 5:**
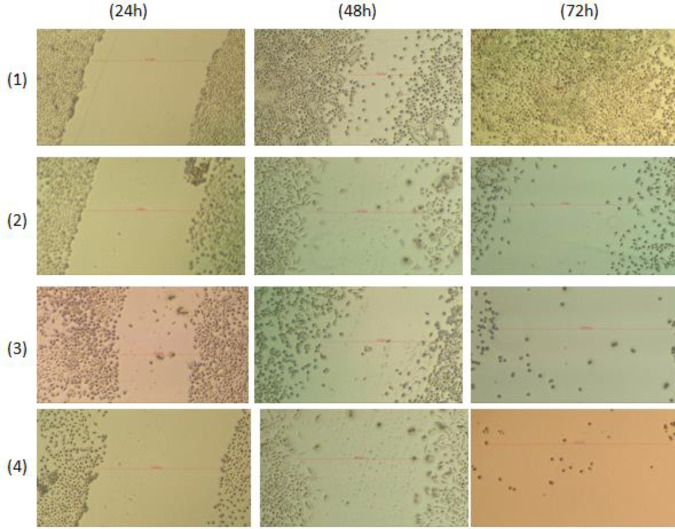
Wound healing in treated groups of 4T1 cells indicates reduction of cellular migration. (1) Control (without any treatment), (2) 1700 μm capecitabine, (3) 3,125 μm mocetinostat, and (4) 50 μm capecitabine, 1,5 μm mocetinostat. Combined treatment of capecitabine and mocetinostat reduced cellular density more than concentrations of mocetinostat and capecitabine applied alone

**Figure 6:**
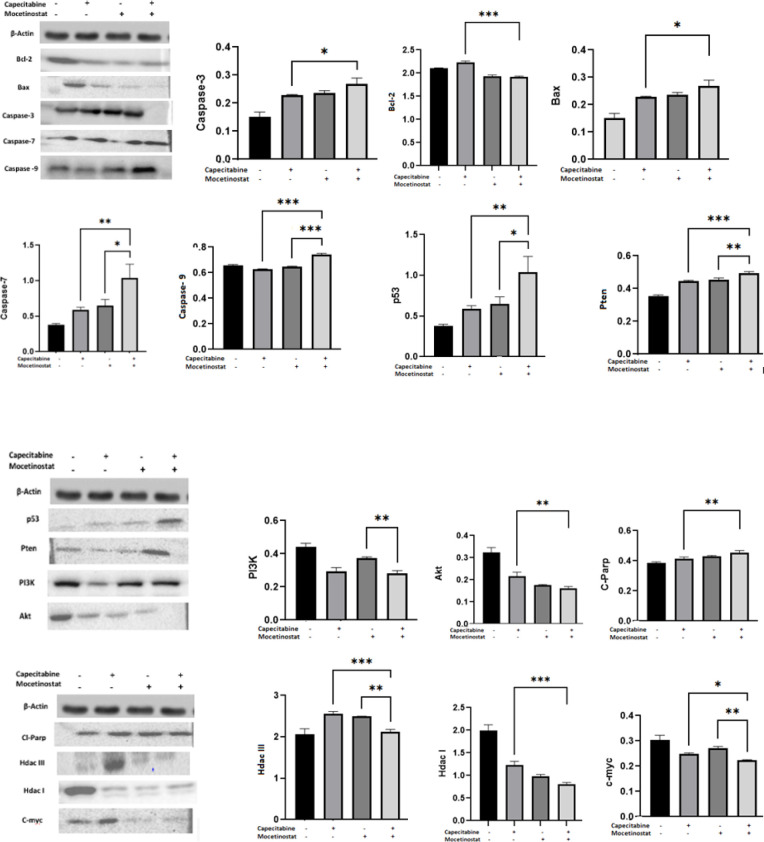
Capecitabine, mocetinostat, capecitabine+mocetinostat induces apoptosis by altering the protein expression of Bcl-2, Hdac I, Akt, Bax, caspase 3, Pten, cleaved-PARP, Caspase-7, Caspase-9, p53, PI3K, c-myc, and Hdac III in 4T1 cells. Bands were quantified by densitometry and normalized to the model control values. A statistical analysis of proteins adjusted to β-actin was performed. The data are shown as the mean standard deviation of three separate experiments. * *P*<0.05, ** *P*<0.01, *** *P*<0.001, respectively

## Discussion

In this study, various methods were used to investigate the antitumor effects on breast cancer 4T1 cells caused by treatment with mocetinostat and capecitabine alone, and treatment by these two drugs combined. 

Combinations of two or more therapeutic drugs, specifically targeting the cancer-causing cell and cell signaling pathways, make important contributions in determining the different mechanisms of drugs (19, 16). Capecitabine may be a favored agent to be evaluated in experimental combination regimens due to its tolerability and efficiency as a ** s**ingle agent and its lack of cross-resistance with other chemotherapeutics (20). Cellular functions such as cell cycle, replication, survival, DNA repair, and differentiation are all regulated by histone deacetylases (HDACs). In hematologic and solid tumors, their expression is often changed (21). A significant number of HDACi are currently in clinical trials as anticancer agents (22). HDACi have less effect to display significant antitumor efficacy as a single agent in solid tumors, including breast cancer, and are more effective when combined with radiotherapy, chemotherapy, or others (23). HDACi reduces cancer cells through apoptosis with the use of many cancer drugs (24). Mocetinostat is well-tolerated in clinical trials, with favorable pharmacokinetics and pharmacodynamics and promising antitumor efficacy in a variety of diseases (25). The 4T1 cell line has immunogenicity, proliferation, and metastatic qualities that are very similar to stage IV human breast cancer. Despite advancements in cancer detection and therapy, new choices are needed to increase survival and enhance the quality of life for these individuals (26).

In the present study, we examined for the first time the effects of capecitabine in combination with mocetinostat on mouse 4T1 cell line. In this study we investigated, the cytotoxic and growth inhibitory effects of Capecitabine, Mocetinostat, Capecitabine+Mocetinostat on 4T1 cancer cells. The viability of 4T1 cells exposed to various capecitabine, mocetinostat, and capecitabine+mocetinostat concentrations was reduced in a concentration and time-dependent manner, according to our findings. On 4T1 cells, the half-maximal inhibition concentrations (IC_50_) were: capecitabine 1700 µM, mocetinostat 3,125 µM, and capecitabine 50 µM+mocetinostat 1,5 µM for 48 hr (Figure 1). 

Zhang *et al*. demonstrated capecitabine alone had the modest inhibitory effect (inhibition rate 35.3 %, data not shown) on 4T1^LUC^ cell line and 3 μg/ml capecitabine, and the survival rates for cells treated for 48 hr in combination with Lidamycin were 63.0, 64.7, and 23.4%, respectively (27). Other studies have shown that IC_50_ values ​​of capecitabine vary from 860 μM to 6000 μM in cancer cells. IC_50_ values ​​of capecitabine were: HCT116 2850 μM, HT29 1590 μM, SW620 4190 μM, HCT8 5957 μM, HCT15 5840 μM, COLO205 863 μM cells for 24 hr (28.). IC_50_ values of mocetinostat on colon carcinoma were: (HCT116) 0.29 μM, HCT15 0.72 μM, HT29 0.76 μM, DU145 0.67 μM, MDA-MB231 0.61 μM, T24 0.66 μM, A549 0.9 μM, BxPc3 1.4 μM, Jurkat-T 0.09 μM, U937 Histiocytic lymphoma 0.11 μM, HL60 Promyelocytic leukemia 0.18 μM, RPMI-8226 0.15 μM, MV-4-11 0.1 μM, HMEC Breast normal epithelial 20 μM, and MRHF Foreskin fibroblasts 15 μM (29). 

Mocetinostat is used combined with another antitumor. Some researchers found that mocetinostat in combination with gemcitabine affects cell growth and induces apoptosis in LMS (Leiomyosarcoma) cells (30, 31). Mocetinostat treatment was 1 μM to 5 μM on DU-145 and PC-3 cells for 72 hr and induced significant levels of apoptosis (32). 

 It has been used in combination with many drugs such as capecitabine, docetaxel and cyclophosphamide, epirubicin, and positive results have been obtained in the treatment of breast cancer (33). In a phase 3 study conducted in 2002, the combined use of docetaxel and capecitabine significantly reduced the risk of disease progression and increased the survival rate in patients (34). Based on literature study, we obtained from the *in vitro* study show that the combined use of mocetinostat and capecitabine is effective on breast cancer cells and induces apoptosis of the cells. 

Different cell viability and proliferation findings were found in our research. 4T1 cells were incubated with capecitabine, mocetinostat and their combination for 24, 48, 72, and 96 hr, and the number of apoptotic cells decreased. Capecitabine+mocetinostat caused 16%, 34.3%, 51.5%, and 88.2 %, respectively for 24, 48, 72 and 96 hr compared with the single uses of capecitabine and mocetinostat. 

Apoptosis was caused by changes in cell morphology in the current investigation. The cytotoxicity was found to be through causing changes in the cell morphology. Cells shrink in volume and lose their cellular extensions. The wound healing area was closed at 72 hr in the control group (*P*<0.05). This study demonstrated the cell migration effect of the combined treatment of capecitabine+mocetinostat. Studies showed that capecitabine and mocetinostat inhibited cell migration (35, 36). Exposing the cells to the combination of capecitabine+mocetinostat, on the other hand, led to a substantial increase in wound area (Figure 5). Capecitabine inhibits DNA synthesis in rapidly proliferating cancer cells and mocetinostat affects apoptosis, but the exact mechanism is unknown (37, 38). One of the methods to determine apoptosis is genomic DNA fragmentation. Single drugs and their combinations triggered DNA fragmentation in 4T1 cells, resulting in a characteristic ladder pattern of apoptotic mechanism, demonstrating apoptosis-mediated cell death. Capecitabine, mocetinostat, and capecitabine+mocetinostat were shown to trigger the apoptotic pathway in the 4T1 cell line. In various cancer models, all HDAC inhibitors have been shown to trigger either an extrinsic or intrinsic cell death pathway, or both together. With co-administration of capecitabine and mocetinostat, the expression of Bcl2, Hdac I, Akt, PI3K, c-myc, and Hdac III, which are active proteins in the apoptotic pathway and cell growth, decreased significantly, while Bax, Cas-3, Pten, C-Parp, Cas-7, Cas-9, p53, and C-cas9 protein expression was significantly increased (Figure 6). It is possible to say that the apoptotic pathway is stimulated by the co-administration of capecitabine and mocetinostat in 4T1 cells by increasing the expression of caspase-3 and caspase-7 proteins involved in the intrinsic pathway. When single and combined use of drugs are evaluated in protein levels, it was shown that capecitabine and mocetinostat affect the apoptotic pathway in single use. While Pten protein level increased with combined drug administration, PI3K protein level decreased and cell proliferation was adversely affected. Combination use times, indicated that drugs are more effective on proteins involved in the apoptotic pathway, and Caspase activity had already risen following treatment. AKT/PIK3 could protect cells from apoptosis and PI3K/Akt protein levels were detected to be decreased when compared with that of control and single drug treatments.

One of the most effective anticancer therapies is the targeted suppression of histone deacetylase (HDAC). In general protein levels of Hdac 1, Hdac 3, and others show high expression on breast cancer (39). We have demonstrated that levels of Hdac I and Hdac III proteins levels were decreased after combined therapy compared with that of both single agents (*P*<0.001). It was observed that HDAC I-3 expression increased in the 4T1 cell line with the co-administration of mocetinostat and capecitabine for 48 hr. It can be said that HDAC inhibitors induce apoptosis in tumor cells with their pro-apoptotic and anti-apoptotic regulation. Some researchers have evaluated the levels of similar and different proteins on different cell lines, but such data are not available for the combined effect of the two drugs on the 4T1 breast cancer cell line (40- 42).

In this study, we report for the first time that using HDACi mocetinostat in conjunction with capecitabine might be a viable option. Mocetinostat in combination with capecitabine revealed increased anti-tumor effects as compared with either treatment alone. 

## Conclusion

In this study, for the first time, we investigated the cytotoxicity of mocetinostat and two drugs combined on the 4T1 cell line. The combination of mocetinostat and capecitabine could significantly inhibit the growth of breast cancer ın vitro and could trigger apoptosis pathways even at very low concentrations. These findings might have a significant impact on breast cancer treatment since significantly lower dosages of capecitabine can result in fewer undesirable side effects. As a result, the combination of mocetinostat and capecitabine may be a novel and effective agent against breast cancer. 

## Authors’ Contributions

OE, HKC Study conception and design; HKC Data processing, collection, performing experiments; OE, HKC Analysis and interpretation of results; OE, HKC Draft manuscript preparation, visualization; OE Critical revision or editing of the article; OE, HKC Final approval of the version to be published; OE, HKC Supervision, funding acquisition.

## Conflicts of Interest

The authors declare that no conflict of interest exists.
